# Supplementary material for: EdgeCaseDNet: An enhanced detection architecture for edge case perception in autonomous driving
Source: PLoS One. 2025 Dec 18;20(12):e0338638. doi: 10.1371/journal.pone.0338638 (PMC12714283; doi:10.1371/journal.pone.0338638)
Supplement: Attachment — Submitted filename: _ PLOS-ONE-Response-to-Reviewers-PONE-D-25-09823.docx [file pone.0338638.s002.docx]

**Original Manuscript ID:** PONE-D-25-09823

**Original Article Title: “**EdgeCaseDNet: An Enhanced Detection Architecture for Edge Case Perception in Autonomous Driving

”

**To:** PLOS ONE Editor

**Re:** Response to reviewers

Dear Dr., Alessio Plebe,

Thank you for your constructive feedback and the opportunity to revise our manuscript "EdgeCaseDNet: An Enhanced Detection Architecture for Edge Case Perception in Autonomous Driving" (ID: PONE-D-25-09823). We have carefully addressed all comments from the reviewers and editors. These comments are all valuable and very helpful for revising and improving our paper, as well as the important guiding significance to our research. Based on these comments and suggestions, we have made appropriate modifications to the original manuscript, then we hope to meet with your approval.

According to your and the reviewers’ comments we have addressed four key areas of improvement:

1. **Implementation of Decision Letter Guidance**
  -*We have meticulously supplemented and revised* the manuscript content in strict accordance with the requirements outlined in your decision letter, ensuring *all feedback has been comprehensively addressed*.

2. **Strengthened Theoretical Rigor**
  - *We have conducted* a comprehensive review of the theoretical framework to enhance its clarity, logical soundness, and alignment with established literature.
  - *We have reinforced* the justification for the assumptions and models through expanded logical derivations and comparative analyses.

3. **Enhanced Experimental Robustness**
  - *We have expanded* the experimental scope.
  - *We have restructured* the Methods section to explicitly detail experimental protocols, evaluation metrics (mAP50-95, FPS, FLOPs), and statistical validation methods.
  - *We have uploaded* the source code to a public repository to ensure full reproducibility.

4. **Refined Academic Presentation**
  - *We have restructured* the manuscript’s narrative flow using transitional phrases and signposting to improve coherence.
We entrusted a professional editing agency to proofread and refine the language.

We are uploading (a) our point-by-point response to the comments (below) (response to reviewers, under “Author’s Response Files*”*), (b) a revised manuscript (track-changes version), and (c) a clean updated manuscript without highlights (“Main Manuscript”*).*

Finally, we appreciate all reviewers’ warm work earnestly and hope that the correction will meet with approval. We are grateful for the opportunity to improve our work through this rigorous review process and look forward to your further guidance.

Best regards,

Sincerely,

Yin Lei

E-mail: 001leiyin@tongji.edu.cn

Corresponding author:

Name: Wancheng Ge

E-mail:[gwc828@tongji.edu.cn](mailto:gwc828@tongji.edu.cn)

Our point-by-point response to the comments (below)：

Journal requirements:

When submitting your revision, we need you to address these additional requirements.

1. Please ensure that your manuscript meets PLOS ONE's style requirements, including those for file naming. The PLOS ONE style templates can be found at

[https://journals.plos.org/plosone/s/file?id=wjVg/PLOSOne_formatting_sample_main_body.pdf](https://track.editorialmanager.com/CL0/https:%2F%2Fjournals.plos.org%2Fplosone%2Fs%2Ffile%3Fid=wjVg%2FPLOSOne_formatting_sample_main_body.pdf/1/010f01967c0aaa2e-5afdbe8f-a155-4496-813d-846db9fb1209-000000/wKUmCZ8dE6_uVTztHL1MXj1c9n9dPVXdVYq3jbnWKUQ=208)  and

[https://journals.plos.org/plosone/s/file?id=ba62/PLOSOne_formatting_sample_title_authors_affiliations.pdf](https://track.editorialmanager.com/CL0/https:%2F%2Fjournals.plos.org%2Fplosone%2Fs%2Ffile%3Fid=ba62%2FPLOSOne_formatting_sample_title_authors_affiliations.pdf/1/010f01967c0aaa2e-5afdbe8f-a155-4496-813d-846db9fb1209-000000/B6iFHz6vAJdd5BP7iUxiVwfk8YNV48yZAIeO9q38Ahs=208)

**Author response:**

We have meticulously revised the entire manuscript in strict accordance with PLOS ONE's style requirements.

2. Please note that PLOS ONE has specific guidelines on code sharing for submissions in which author-generated code underpins the findings in the manuscript. In these cases, we expect all author-generated code to be made available without restrictions upon publication of the work. Please review our guidelines at https://journals.plos.org/plosone/s/materials-and-software-sharing#loc-sharing-code and ensure that your code is shared in a way that follows best practice and facilitates reproducibility and reuse.

**Author response:**

We have archived all pertinent code in an open software repository, accessible at https://github.com/yutianku/EdgeCaseDNet.

3. Thank you for stating the following financial disclosure:

“This research was supported in part by the International Strategic Innovative Project of the National Key Research and Development Program of China under Grant(No. 2023YFE0112500)”

Please state what role the funders took in the study.  If the funders had no role, please state: "The funders had no role in study design, data collection and analysis, decision to publish, or preparation of the manuscript."

If this statement is not correct you must amend it as needed.

Please include this amended Role of Funder statement in your cover letter; we will change the online submission form on your behalf.

**Author response:**

We have updated the Role of Funder statement in our cover letter to meet the requirements.

4. Thank you for stating the following in the Acknowledgments Section of your manuscript:

“This research was supported in part by the International Strategic Innovative Project of the National Key Research and Development Program of China under Grant(No. 2023YFE0112500)”

We note that you have provided funding information that is currently declared in your Funding Statement. However, funding information should not appear in the Acknowledgments section or other areas of your manuscript. We will only publish funding information present in the Funding Statement section of the online submission form.

Please remove any funding-related text from the manuscript and let us know how you would like to update your Funding Statement. Currently, your Funding Statement reads as follows:

“This research was supported in part by the International Strategic Innovative Project of the National Key Research and Development Program of China under Grant(No. 2023YFE0112500)”

Please include your amended statements within your cover letter; we will change the online submission form on your behalf.

**Author response:**

We have removed the funding-related text from the manuscript in accordance with your instructions. The Funding Statement will remain unchanged as "This research was supported in part by the International Strategic Innovative Project of the National Key Research and Development Program of China under Grant (No. 2023YFE0112500)." No further amendments are needed.

5. When completing the data availability statement of the submission form, you indicated that you will make your data available on acceptance. We strongly recommend all authors decide on a data sharing plan before acceptance, as the process can be lengthy and hold up publication timelines. Please note that, though access restrictions are acceptable now, your entire data will need to be made freely accessible if your manuscript is accepted for publication. This policy applies to all data except where public deposition would breach compliance with the protocol approved by your research ethics board. If you are unable to adhere to our open data policy, please kindly revise your statement to explain your reasoning and we will seek the editor's input on an exemption. Please be assured that, once you have provided your new statement, the assessment of your exemption will not hold up the peer review process.

**Author response:**

We agree to make the code publicly available. All relevant code has been uploaded to GitHub and can be accessed at <https://github.com/yutianku/EdgeCaseDNet>.

**Reviewer#1, Concern # 1 (Theoretical clarity on HGNetv2 vs RT-DETR. The manuscript incorrectly refers to HGNetv2 as the "RT-DETR backbone network" (e.g., Lines 50–51, 125–126). This is misleading—HGNetv2 is a CNN backbone that can be used within models like RT-DETR, but it is not part of RT-DETR’s transformer-based architecture. This conceptual confusion weakens the theoretical grounding and should be corrected throughout the manuscript to accurately reflect HGNetv2’s role as a standalone CNN backbone integrated with YOLOv8 in this work.):**

**Author response:**

We sincerely appreciate your insightful comment regarding the theoretical clarity of HGNetv2 and its relationship to RT-DETR. You are correct that referring to HGNetv2 as the "RT-DETR backbone network" is inaccurate and may lead to conceptual confusion. Through further study, we have come to understand that the HGNetv2 is indeed a CNN-based backbone that can be optionally integrated into various detection frameworks, including RT-DETR, but it is not inherently part of RT-DETR’s transformer-based architecture.

**Author action:**

We have carefully reviewed the manuscript to correct all instances where HGNetv2 was improperly referred to as part of RT-DETR. Specifically, in Lines 48,140 and 420, we have revised the text to clarify that HGNetv2 is a standalone CNN backbone and that, in this work, it is integrated with YOLOv8 rather than RT-DETR. These changes have been applied consistently throughout the manuscript to enhance theoretical accuracy and avoid misinterpretation.

**Reviewer#1, Concern # 2 (Figure 1 notation and clarification. The manuscript refers to “dot lines” in Figure 1, but the lines are red dashed lines. The terminology should be corrected for clarity. Additionally, multiple red dashed boxes are used—each box’s function should be clearly labeled or described in the caption.):**

**Author response:**

Thank you for your careful observation regarding the notation and clarity of Figure 1. We agree that this inconsistency could lead to confusion and appreciate your suggestion to improve the description of the figure. Furthermore, we acknowledge the need to provide clearer labels or descriptions for the multip^[1]^le red dashed boxes shown in the figure.

**Author action:**

We have updated the notation and clarification of Figure 1 from “dot lines” to “red dashed lines” to accurately reflect the visual elements presented in Figure 1. Additionally, we have revised the caption of Figure 1 to include detailed descriptions of each red dashed box, explaining their respective functions. This will ensure that readers can easily understand the purpose and significance of each component within the figure, enhancing overall clarity and readability.

Revised Text:

**Fig 1. The architectural framework of EdgeCaseDNet.** The red dashed lines box shows the optimized elements of the improved network structure. For a detailed examination of the HPDSConv module, please refer to Fig 3.

**Reviewer#1, Concern # 3 (Figure 2 – incomplete HG-block architecture. Figure 2, titled "The Network Architecture of HG-Block," fails to show the architecture in sufficient detail. Modules such as “CBA[4]”, “CBA[5]”, etc., appear without any explanation. Please define what ConvBAct (CBA) means and describe each block’s function. A clear legend and detailed block-level diagram are recommended.):**

**Author response:**

Thank you for highlighting the lack of detail in the depiction of the HG-block architecture in Figure 2. You are correct that simply presenting modules such as “CBA[4]”, “CBA[5]”, etc., without adequate explanation can lead to confusion and does not provide enough insight into the functionality of each component within the HG-block.

**Author action:**

We have taken several steps to improve the clarity and comprehensiveness of Figure 2. Firstly, we have defined ConvBAct (CBA) as a module consisting of a convolutional layer followed by batch normalization and activation function, typically used to process feature maps efficiently within neural networks. We have added this definition along with brief descriptions of each block’s function directly into the figure caption.

Additionally,  we have also developed a clear, block-level diagram of the HG-block architecture, which breaks down each module and clearly shows their interconnections and functions. These changes aim to make Figure 2 more informative and accessible, ensuring that readers can fully grasp the architecture and design of the HG-block.

Revised Text:

**Fig 2. The Network Architecture of HG-Block.** In the HG-Block architecture, the ESE module is integrated into the network structure to enhance feature extraction and representation capabilities. The  Convolution-BatchNorm-Activation( ConvBAct module) is composed of a convolutional layer (Conv), batch normalization layer (BatchNorm), and activation function (Activation). It serves as a fundamental building block for feature extraction and feature transformation in deep neural networks.

**Reviewer#1, Concern # 4 (Equation symbols. Several equations throughout the manuscript use variables (e.g., , , , , , etc.) without proper definitions or descriptions. All symbols should be explicitly defined when first introduced to ensure clarity and reproducibility.):**

**Author response:**

Thank you for pointing out the issue regarding the undefined symbols in the equations.

**Author action:**

We have thoroughly reviewed all equations throughout the manuscript and added clear definitions for each variable at the point of first use.

**Reviewer#1, Concern #5 (Figure 4 – Visual overlap issue. In Figure 4, the overlay of text and image elements reduces legibility. Please adjust the layout to ensure all components (e.g., arrows, letters, and labels) are clearly visible and non-overlapping.):**

**Author response:**

Thank you for your valuable feedback regarding the visual clarity of Figure 4. We agree with your suggestions and have incorporated the recommended changes into the manuscript.

**Author action:**

To address this issue, we have revised the layout of Figure 4 to improve the visibility and clarity of all graphical and textual elements. Specifically, we have repositioned labels and adjusted the placement of arrows to avoid any overlap with critical parts of the images.

Revised Text:

**Fig 4 . Structure of the AFPN**

**Reviewer#1, Concern # 6 (Check the usage of the language.):**

**Author response:**

Thank you for your suggestion to check the language usage throughout the manuscript. We recognize the importance of linguistic accuracy in ensuring that our work is accessible and easily understood by a broad academic audience.

**Author action:**

We have carefully reviewed the entire manuscript to thoroughly examine the language usage. In addition, we have engaged a professional English editing service to perform a comprehensive proofreading and revision of the text. This process included correcting any grammatical errors, improving sentence structure for clarity and fluency, and ensuring consistency in terminology and style throughout the paper. All revised sections have been double-checked to maintain the original technical meaning while enhancing readability and coherence.

Thanks for your thoughtful suggestion. Your comments are all valuable and very helpful for revising and improving our paper, as well as the important guiding significance to our research.

**Reviewer#2, Concern # 1 (While there is an ablation study, it could benefit from a more detailed analysis of why each component contributes to the observed improvements.):**

**Author response:**

Thank you for your insightful comment regarding the ablation study. We agree with your suggestions and have incorporated the recommended changes into the manuscript.

**Author action:**

To ensure the effective presentation of these new additions, we have taken the following measures:

1）In the **ablation study experiment** section, we supplemented an in- depth analysis of the ablation experiment results of the Wise - IoU loss function, HarrHGNetv2, and the AFPN module. Meanwhile, we used figures for visual presentations.

Revised Text:

To address these issues, we introduce a novel bounding box regression loss function named **Wise-IoU**, inspired by the geometric properties of axis-aligned rectangles.

2）In the **Methods** section, we elaborated and updated the theoretical support behind each modification, explaining why specific design choices could lead to performance improvements.

Revised Text :

1) Aliasing suppression: Strict band limiting via orthogonal basis functions reduces spectral leakage

2)Feature persistence: Multiscale representations are preserved through explicit sub band decomposition

3) Differentiability: The linear transform is implemented as a learnable-compatible matrix multiplication, allowing end-to-end training.  Meanwhile, utilizing fixed wavelet kernels, this approach demonstrates greater computational efficiency compared to convolutional layers, as it eliminates the need for training parameters and reduces computational demands.

**Reviewer#2, Concern # 2 (The construction of the DEAP dataset would strengthen the paper, particularly regarding how edge cases were identified and selected.):**

**Author response:**

Thank you for your valuable comment regarding the construction of the DEAP dataset. You are right that a more detailed explanation of how edge cases were identified and selected would significantly enhance the credibility and transparency of our work. Clarifying this process not only supports reproducibility but also demonstrates the robustness and generalization capability of our model in challenging scenarios.

**Author action:**

In section “**4.1 Dataset & Experimental Environment”**, we have updated the description concerning the construction of the DEAP dataset as follows:

Revised Text:

First, the dataset includes four typical adverse weather conditions and extreme driving scenarios, integrating over 2,000 edge case scenarios through a multi-source data fusion strategy. Some samples were selected from the authoritative open-source datasets CODA and SODA10M and subjected to rigorous quality screening and scenario validation. Second, DEAP features a structured data system with 13,030 annotated samples, which were divided into training (9,121 samples), validation (2,606 samples), and testing (1,303 samples) sets in a 7:2:1 ratio. Then we use data augmentation ( rotation, scaling, partial occlusion, etc. ) to expand the data set, and finally use the semi-automatic annotation tool X-AnyLabeling[] to annotate the image data.

**Reviewer#2, Concern # 3 (While the authors mention reducing parameters and computational complexity throughout the paper, it lacks an in-depth computational analysis. A more comprehensive analysis of inference time and computational requirements would strengthen the practical applicability claims.):**

**Author response:**

Thank you for your constructive feedback regarding the computational analysis in our paper. You are correct that while we have mentioned reductions in model parameters and computational complexity, a more in-depth and quantitative evaluation of inference time and computational requirements is essential to substantiate our claims regarding practical applicability and efficiency.

**Author action:**

In response, we have made substantial additions and improvements in both the methodology and experimental sections of the paper.

In **Section 3 – Methods**, we have added *theoretical analysis of model lightweighting* to provide a detailed explanation of how the proposed modules contribute to reducing model complexity while preserving performance. Specifically:

1. We introduced the **Haar_HGNetV2 module**, which replaces the traditional downsampling operations in HGNetv2 with **Haar wavelet-based downsampling (HWD)**. This method preserves more structural information during downsampling while reducing aliasing effects, thereby achieving better feature representation with comparable or even reduced computational cost.
2. We proposed a novel **Lightweight Hybrid Partial Depth-Wise Separable Convolution (HPDSConv) module**, which integrates depth-wise separable convolution with partial convolution techniques. This design significantly reduces the number of parameters and FLOPs by decomposing standard convolutions into depth-wise and point-wise operations, while maintaining effective feature extraction through the hybrid structure.

In addition, in **Section 4.5 – Ablation Study**, we have added experimental results that quantitatively evaluate the computational efficiency of the model, including:

- **Parames**
- **GFLOPs** (Giga Floating Point Operations) for each variant of the model;

These results clearly demonstrate the effectiveness of the Haar_HGNetV2 and HPDSConv modules in reducing computational load, supporting our claims regarding model lightweighting and practical deployment potential.

**Reviewer#2, Concern #4 (4. The manuscript contains numerous grammatical errors and awkward phrasings that impede comprehension. For example:
4.1 Line 74-75: "These methods are cumbersome, have poor generalization and robustness, and cannot meet practical road-scenario detection requirements." This is an overgeneralization without supporting evidence.
4.2 Line 149-151: "The Tiny model contains only three LDS Layers to maximize the GPU computation efficiency." The causal relationship is not clearly explained.
4.3 Line 238-239: " ∈[1, e) increases the focus on anchors of average quality." The meaning of "anchors of average quality" is not defined.):**

**Author response:**

Thank you for your meticulous review and for pointing out specific instances where the language and clarity of our manuscript could be improved. Addressing these issues will undoubtedly enhance the readability and professionalism of our work. Below are detailed responses to each point raised:

**Author action:**

**Point 4.1**

Original Text (Lines 74-75): "These methods are cumbersome, have poor generalization and robustness, and cannot meet practical road-scenario detection requirements."

Issue: Overgeneralization without supporting evidence.

Action Taken: We have revised this statement to provide a more balanced perspective and included references to empirical studies or examples that support our claim.

Revised Text:

These traditional object detection algorithms rely on handcrafted features[1], they may struggle with scale variations, occlusion, and dynamic lighting conditions in complex traffic scenarios. Nonetheless, these approaches established a foundational framework for advancing subsequent detection methodologies [2].

[1]Z. Zou, K. Chen, Z. Shi, Y. Guo, J. Ye, Object Detection in 20 Years: A Survey, Proc. IEEE 111 (2023) 257–276. https://doi.org/10.1109/JPROC.2023.3238524.

[2]L. Jiao, R. Zhang, F. Liu, S. Yang, B. Hou, L. Li, X. Tang, New Generation Deep Learning for Video Object Detection: A Survey, IEEE Trans. Neural Netw. Learning Syst. 33 (2022) 3195–3215. https://doi.org/10.1109/TNNLS.2021.3053249.

**Point 4.2**

Original Text (Lines 149-151): "The Tiny model contains only three LDS Layers to maximize the GPU computation efficiency."

Issue: Lack of clear explanation regarding the causal relationship.

Action Taken: We have clarified the rationale behind choosing exactly three LDS layers and how it impacts GPU computation efficiency.

Revised Text:

"The Tiny model employs precisely three LDS (Lightweight DownSampling) layers. This design choice strikes an optimal balance between reducing computational load and maintaining effective feature extraction, thereby maximizing GPU computation efficiency."

**Point 4.3**

Original Text (Lines 238-239): " ∈[1, e) increases the focus on anchors of average quality."

Issue: Undefined term "anchors of average quality".

Action Taken: We have defined what constitutes "anchors of average quality" and provided context for how varying the parameter influences model performance.

Revised Text:

"Adjusting the parameter within the range ∈[1, e) increases the model's emphasis on anchors that exhibit moderate confidence scores, referred to as 'anchors of average quality'. These anchors typically represent regions of interest that are neither highly certain nor extremely uncertain, thus balancing precision and recall during training."

**Reviewer#2, Concern #5 (The term "edge case" is central to the paper, but it is never precisely defined. What constitutes an edge case versus a normal case should be clearly stated.):**

**Author response:**

Thank you for your thoughtful suggestion. You are absolutely right that although we have mentioned challenging detection conditions in both the Introduction and Dataset & Experimental Environment sections, a more explicit and unified definition of *edge case* is necessary—especially given its central role in motivating our work and evaluating model robustness.

**Author action:**

To clearly emphasize the newly proposed concept of “edge case” and ensure consistency throughout the paper, we have revised the **Introduction section** to include a more focused clarification. Specifically:

- We have added a concise yet precise definition of *edge case*, aligned with the descriptions provided in Section 4.1, while also linking it directly to the motivation behind constructing the DEAP dataset.
- We have contrasted *edge cases* with *normal cases* to further enhance clarity and help readers understand the practical implications of detecting such samples in real-world road scenarios.

**Revised Paragraph (Introduction Section):**

"In real-world road scenarios, object detection models often encounter *edge cases*—situations where detection becomes exceptionally challenging due to factors such as severe occlusion, adverse weather conditions (e.g., fog, rain, snow), low illumination, small object size, or complex background interference. These cases deviate from the typical distribution of training data and can lead to high rates of false negatives and positives, posing serious safety risks in autonomous driving systems. To address this critical issue, we propose the DEAP dataset, specifically designed to evaluate model performance under such demanding conditions."

This revision ensures that the term *edge case* is introduced early and clearly, reinforcing its importance and laying a solid foundation for the rest of the paper. Thank you again for your insightful feedback.

**Reviewer#2, Concern #6 (6. In Section 3.2, "Partial Convolution (PConv)" is introduced, but its mathematical formulation is missing, unlike that of other components.):**

**Author response:**

Thank you for your careful review and for pointing out the omission of the mathematical formulation for **Partial Convolution (PConv)** in Section 3.2. For consistency and completeness, all core components—including PConv—should be accompanied by a clear mathematical description to facilitate understanding and reproducibility.

**Author action:**

We have added the mathematical formulation of Partial Convolution (PConv) in Section 3.2 ("Partial Convolution (PConv)") to align it with the level of detail provided for other modules in the paper. The revised section now includes:

A formal definition of how partial convolution operates differently from standard convolution, particularly in terms of dynamic feature updating based on valid input regions.

The corresponding mathematical equations that describe the computation process, including mask handling and feature re-normalization.

Revised Content (Section 3.2):

4) Pointwise convolution

Pointwise convolution was used to combine the outputs of the deep convolution. For deep convolution, the output feature map $( D )$ has dimension$(H^{'}\times W^{'}\times C_{1})$, the convolution kernel $( W )$using $( 1 \times1 )$ with dimensions $(1\times1\times C_{1}\times C_{2})$ and the output feature map $( P )$ has dimensions $(H^{'}\times W^{'}\times C_{2})$ where$(C_{2})$ is the number of output channels. The output of the point-by-point convolution can be calculated using Eq (4).

$[P\left( m,n,k \right)=\sum_{c} D\left( m,n,c \right)\cdot W\left( 1,1,c,k \right)]$	(4)

where $( k )$ is the output channel index. Point-by-point convolution is a linear combination of feature maps of each channel to produce a new channel.

Batch normalization normalizes each feature channel with the following Eq (5).

$\left\{ \begin{aligned} [\hat{x}=\frac{x-\mu}{\sqrt{\sigma^{2}+\epsilon}}] \\ [y=\gamma\hat{x}+\beta] \end{aligned} \right.$	(5)

where $( x )$ is the input, $( \mu)$ and $(\sigma^{2})$ are the mean and variance of the channel, respectively, $( \epsilon)$ is a small number to avoid division by zero, and $( \gamma)$ and $( \beta)$ are learnable parameters.

The mathematical expression for the SiLU (Sigmoid Linear Unit) activation function is shown in Eq (6).

$[\text{SiLU}\left( x \right)=x\cdot\sigma\left( \alpha x \right)]$	(6)

where $( \sigma)$ is a sigmoid function and $( \alpha)$is a learnable parameter or a fixed constant.

**Reviewer#2, Concern #7 (The paper alternates between "HPDSCM" and "HPDSConv" when referring to the same module.):**

**Author response:**

Thank you for catching the inconsistency in terminology regarding the module referred to as both "HPDSCM" and "HPDSConv" throughout the paper. Consistent terminology is crucial for clarity and readability, ensuring that readers are not confused by varying abbreviations or names for the same concept.

**Author action:**

To address this issue, we have standardized the naming convention for the **Hybrid Partial Depth-Wise Separable Convolution Module** across the entire manuscript. We will adopt "HPDSConv" as the standard abbreviation since it more clearly reflects the nature of the module and its function. Here’s what we’ve done:

**1) Global Search and Replace:** We conducted a thorough review of the manuscript to identify all instances where either "HPDSCM" or "HPDSConv" was used. All occurrences of "HPDSCM" have been replaced with "HPDSConv" to maintain consistency.

**2) Clarification in Definitions:** In the initial introduction of the HPDSConv module, we have clarified its full name and abbreviation to avoid any confusion:

Example: "We introduce the **Hybrid Partial Depth-Wise Separable Convolution (HPDSConv)** module, designed to reduce computational complexity while preserving model performance."

**3) Consistency Check:** We have ensured that all figures, tables, and legends referring to this module use the consistent term "HPDSConv."

By making these adjustments, our goal is to ensure that the terminology is uniform throughout the document, enhancing readability and comprehension. We appreciate your attention to detail, which has helped us improve the quality of our manuscript.

**Minor changes:**

**Reviewer#2, Concern #1 (The description of performance improvements appears in nearly identical form in the abstract, introduction, and conclusion.):**

**Author response:**

Thank you for your thoughtful observation regarding the repetition of performance improvement descriptions across the abstract, introduction, and conclusion. We agree with your suggestions and have incorporated the recommended changes into the manuscript.

**Author action:**

We have carefully revised the manuscript to ensure that:

**1) Abstract:** Now provides a concise summary of the key contributions and overall performance improvements without going into detailed comparisons or explanations.

Autonomous driving perception systems still encounter significant challenges in edge scenarios involving multi - scale target changes and adverse weather, which seriously compromises detection reliability.To address this issue, we introduced a novel edge-case dataset which extending a benchmark that captures extreme road conditions (fog, rain, snow, nighttime) with precise annotations and developed EdgeCaseDNet, an optimized object-detection framework. The EdgeCaseDNet’s architecture extends YOLOv8 through four synergistic innovations: (1) a Haar_HGNetv2 backbone that enables hierarchical feature extraction with enhanced long-range dependencies; (2) an asymptotic feature pyramid network for context-aware multi-scale fusion; (3) a hybrid partial depth-wise Separable Convolution module; and (4) Wise-IoU loss optimization for accelerated convergence. Comprehensive evaluations demonstrated the superiority of EdgeCaseDNet over YOLOv8, achieving improvements of +1.3% in precision, +7.78% in recall, and +5.91% in mAP50-95. All relevant code are available at [https://github.com/yutianku/EdgeCaseDNet](https://github.com/yutianku/EdgeCaseDNet.git)

**2) Introduction:** Focuses on setting the research context, identifying limitations in existing methods, and clearly stating the motivation and novelty of our approach. The performance results are briefly mentioned to support the significance of the work but not repeated verbatim from the abstract.

To address these limitations, this study makes two major contributions.
(1) The **DEAP dataset** is a new benchmark specifically designed for evaluating object detection models under extreme road conditions. Built upon data from SODA10M[7] and CODA[8] and enriched through advanced augmentation techniques, DEAP provides a realistic and comprehensive testbed for assessing the robustness and generalization of vision-based perception systems.
(2) An improved detection architecture called **EdgeCaseDNet** was built on the YOLOv8 framework. EdgeCaseDNet incorporates several novel components: a **Haar_HGNetv2** backbone inspired by PP-HGNet[] for better feature representation, an **AFPN** module for enhanced multiscale fusion, a **lightweight HPDSConv convolution module** to reduce computational load, and the **Wise-IoU**[6] **loss function** to improve regression accuracy and convergence behavior.

Together, these innovations enable EdgeCaseDNet to deliver higher accuracy and robustness under challenging real-world conditions, while maintaining an efficient inference speed. This study aims to advance the development of safer and more reliable perception systems for autonomous vehicles operating in complex environments.

**3)Conclusion:** Highlights the broader implications of our findings, summarizes the technical contributions, and reflects on the practical benefits of our method—while still referencing the performance improvements as validation, but expressed in a different tone and structure than in the earlier sections.

**Reviewer#2, Concern #2 (The explanation of the CIoU loss function's limitations is repeated in similar terms in lines 229-232 and again when introducing the Wise-IoU loss.):**

**Author response:**

Thank you for pointing out the repetition in the explanation of the limitations of the CIoU loss function. We agree with your suggestions and have incorporated the recommended changes into the manuscript.

**Author action:**

We have revised the **Loss Function** section to eliminate redundant descriptions of CIoU’s limitations. Specifically:

**1) We retained the initial detailed explanation** of CIoU’s limitations as it sets up the motivation for the proposed method.

**2) We removed the repetitive summary** of these limitations when introducing the Wise-IoU loss.

**3) We replaced the original sentence:**

"Considering the shortcomings of the CIoU loss function, a new bounding box regression loss function, Wise-IoU, inspired by the geometric features of horizontal rectangles, was introduced to replace CIoU."

**with the suggested improved version:**

"To address these issues, we introduce a novel bounding box regression loss function named Wise-IoU, inspired by the geometric properties of axis-aligned rectangles."

**Reviewer#2, Concern #3 (The references to the figures are inconsistent in the manuscript. "Figure 1 shows the...." should be "Fig. 1. shows the....):**

**Author response:**

Thank you for bringing to our attention the inconsistency in figure reference formatting throughout the manuscript. We have carefully reviewed all figure references in the text and standardized them according to the journal’s or conference’s formatting guidelines.

**Author action:**

To address this issue, we have:

1) **Replaced all instances of "Figure" with "Fig."** when followed by a number within the body of the text (e.g., changing “Figure 1” to “Fig. 1”).
2) **Ensured that each figure reference ends with a period after the figure number**, following the format: **“Fig. X.”**
   Example:
   - Original: *Figure 1 shows the*  structure of the modified algorithm.
   - Revised: *Fig. 1 shows the* structure of the modified algorithm.
3) **Checked captions of all figures** to ensure they also follow the same format.
4) **Performed a global search** for any remaining inconsistencies using find-and-replace tools to ensure no instance was overlooked.

These revisions guarantee uniformity in figure referencing throughout the manuscript, improving readability and adherence to formatting standards.

**Reviewer#2, Concern #4 (Figure 1 is cluttered, especially the HPDSCM, which is quite small and difficult to follow):**

**Author response:**

Thank you for your guidance on improving the clarity and readability of our manuscript. In response to the feedback regarding the crowded presentation of Figure 1, particularly the difficulty in following the details of the HPDSCM module, we have made targeted revisions. Specifically, we have optimized Figure 1 and adjusted its caption to direct readers to Figure 3 for a detailed view of the HPDSConv module.

**Author action:**

To address the noted issues, we have taken the following steps:

1) **Optimized Figure 1**:
   - We have streamlined the layout of Figure 1 to reduce visual clutter, ensuring that each component is clearly distinguishable.
   - Added comprehensive annotations within the figure to guide the reader through the architecture more effectively.
2) **Updated Caption of Figure 1**:
   - The caption of Figure 1 now includes a reference directing readers to consult Figure 3 for a detailed breakdown of the HPDSConv module. This change ensures that those interested in the specifics of this module can easily find more detailed information without overwhelming Figure 1 with too much detail.

Thanks for your thoughtful suggestion. Your comments are all valuable and very helpful for revising and improving our paper, as well as the important guiding significance to our research.
