# Supplementary material for: EdgeCaseDNet: An enhanced detection architecture for edge case perception in autonomous driving
Source: PLoS One. 2025 Dec 18;20(12):e0338638. doi: 10.1371/journal.pone.0338638 (PMC12714283; doi:10.1371/journal.pone.0338638)
Supplement: Attachment — Submitted filename: _ PLOS-ONE-Response-to-Reviewers-PONE-D-25-09823R1.docx [file pone.0338638.s003.docx]

**Original Manuscript ID:** PONE-D-25-09823**R1**

**Original Article Title: “**EdgeCaseDNet: An Enhanced Detection Architecture for Edge Case Perception in Autonomous Driving”

**To:** PLOS ONE Editor

**Re:** Response to reviewers

Dear Editor,

Thank you for your constructive feedback and the opportunity to revise our manuscript "EdgeCaseDNet: An Enhanced Detection Architecture for Edge Case Perception in Autonomous Driving" (ID: PONE-D-25-09823R1). We have carefully addressed all comments from the reviewers and editors. These comments are all valuable and very helpful for revising and improving our paper, as well as the important guiding significance to our research. Based on these comments and suggestions, we have made appropriate modifications to the original manuscript, then we hope to meet with your approval.

According to your and the reviewers’ comments we have addressed four key areas of improvement:

1. **Implementation of Decision Letter Guidance**

  -*We have meticulously supplemented and revised* the manuscript content in strict accordance with the requirements outlined in your decision letter, ensuring *all feedback has been comprehensively addressed*.

2. **Strengthened Theoretical Rigor**

  - *We have conducted* a comprehensive review of the theoretical framework to enhance its clarity, logical soundness, and alignment with established literature.
  - *We have reinforced* the justification for the assumptions and models through expanded logical derivations and comparative analyses.

3. **Enhanced Experimental Robustness**

  - *We have expanded* the experimental scope.

  - *We have restructured* the Methods section to explicitly detail experimental protocols, evaluation metrics (mAP50-95, FPS, FLOPs), and statistical validation methods.

  - *We have uploaded* the source code to a public repository to ensure full reproducibility.

4. **Refined Academic Presentation**

  - *We have restructured* the manuscript’s narrative flow using transitional phrases and signposting to improve coherence.

We entrusted a professional editing agency to proofread and refine the language.

We are uploading (a) our point-by-point response to the comments (below) (response to reviewers, under “Author’s Response Files*”*), (b) a revised manuscript (track-changes version), and (c) a clean updated manuscript without highlights (“Main Manuscript”*).*

Finally, we appreciate all reviewers’ warm work earnestly and hope that the correction will meet with approval. We are grateful for the opportunity to improve our work through this rigorous review process and look forward to your further guidance.

Best regards,

Sincerely,

Yin Lei

E-mail: 001leiyin@tongji.edu.cn

Corresponding author:

Name: Wancheng Ge

E-mail:[gwc828@tongji.edu.cn](mailto:gwc828@tongji.edu.cn)

**Reviewer#3, Concern # 1 (The core contributions are primarily combinations of existing techniques rather than fundamental innovations. Haar wavelets have been used in computer vision for decades, AFPN and Wise-IoU are from prior work, and HPDSConv appears to be a straightforward combination of depthwise separable and partial convolutions. The authors are encouraged to clarify their contributions more clearly.):**

**Author response:**

We greatly value your insightful feedback. You are indeed correct in noting that the individual components utilized in EdgeCaseDNet, such as Haar wavelets, AFPN, Wise-IoU, and HPDSConv, are derived from or inspired by existing literature. Our objective was not to assert fundamental algorithmic innovation within each module but rather to illustrate how the deliberate integration of these well-established techniques can result in substantial performance improvements, particularly for the complex issue of edge case perception in autonomous driving.

**Author action:**

In the revised manuscript, we will reframe the contribution statement in Sections 1 (Introduction)  to emphasize that our primary innovation lies in the strategic integration and domain-specific adaptation of these components into a cohesive architecture tailored for rare and ambiguous driving scenarios. We will explicitly acknowledge the origins of each technique and clarify that the novelty emerges from their synergistic combination and empirical validation under edge-case conditions rather than from the introduction of new atomic operations.

**Revised manuscript text (Section1: Introduction)**

”The primary contribution of this study lies in the domain-adaptive integration and joint optimization of these components to effectively address the characteristic challenges in autonomous driving edge cases, such as the presence of rare objects, ambiguous scene backgrounds, and low signal-to-noise ratios. “

As clearly articulated in the Introduction, we explicitly delineate the distinction between our approach and existing methods to prevent any misinterpretation of our technical contribution.

**Reviewer#3, Concern # 2 (The DEAP dataset claims comprehensiveness with only 13,030 samples, which seems insufficient for robust edge case representation. The selection criteria from SODA10M and CODA are not clearly explained.):**

**Author response:**

Thank you for raising this important concern regarding the scale of the DEAP dataset and the clarity of the selection criteria for SODA10M and CODA.

Regarding the dataset size, although the original DEAP contained 13,030 samples, it was never intended to compete with large-scale general-purpose datasets such as BDD100K. Instead, DEAP is a carefully curated benchmark focused specifically on high-impact edge cases in autonomous driving perception.

**Author action:**
To further strengthen the scale and environmental diversity of DEAP, we expanded the dataset by integrating 1,027 real-world traffic images from the DAWN dataset, which captures challenging conditions across a wide spectrum of weather (e.g., fog, snow, heavy rain, and sand). This brings the total size of DEAP to 14,057 annotated images, enhancing its coverage of adverse, real-world driving environments.

**Revised manuscript text (Section**4.1**:** Dataset and Experimental Environment**):**

“All samples have been manually verified to exhibit at least one of the following characteristics: (i) extremely rare object categories, (ii) severe occlusion, motion blur, or extreme scale variation, or (iii) adverse environmental conditions (e.g., heavy rain, fog, snow, low illumination) that lead to low signal-to-noise ratios in visual perception.”

This expansion not only increases the dataset scale but also improves the realism and ecological validity of our edge-case evaluation, directly addressing concerns regarding representativeness and generalizability.

**Reviewer#3, Concern # 3 (The 7:2:1 split is standard, but potential class imbalance (e.g., few tricycles) may bias results. Table 1 lists only a single “train/val number” per class (e.g., Car 49,771 vs Tricycle 346), which suggests severe imbalance yet no stratification strategy, per-condition counts (night/rain/fog/snow), or per-split class statistics are reported. Supply train/val/test counts per class and per condition, plus sampling policies to avoid leakage.):**

**Author response:**

Thank you for raising this important concern regarding emphasizing the critical importance of dataset balance, stratification, and transparency in benchmark design. You are correct that the pronounced class imbalance in DEAP may introduce evaluation bias if not rigorously controlled. Our initial presentation reported aggregate per-class counts but omitted essential details regarding split-level distributions and condition-wise coverage, thereby limiting the interpretability and reproducibility of our results.

**Author action:**

We have substantially enhanced the dataset documentation in the revised manuscript as follows:

1) **Per-split class statistics**: We introduce **Table 3**, which reports the exact number of annotated instances for each object category across the training, validation, and test sets, adhering to an approximate 70:20:10 ratio while preserving representation of rare classes.

**Table 3. Per-class train/val/test instance counts in DEAP dataset**

| Object Class | Train | Val | Test | Total |
| --- | --- | --- | --- | --- |
| Image Amount | 9837 | 2809 | 1411 | 14057 |
| Car | 42591 | 11905 | 6114 | 60610 |
| Truck | 10828 | 3097 | 1372 | 15297 |
| Bus | 2493 | 728 | 350 | 3571 |
| Bicyclist | 7377 | 2034 | 1085 | 10496 |
| Pedestrian | 7355 | 2053 | 1036 | 10444 |
| Motorcycle | 349 | 71 | 41 | 461 |
| Total | 70993 | 19888 | 9998 | 100879 |

2) **Per-condition × per-class breakdown**: A new cross-tabulation (**Table 4**) details instance counts for every object class under five distinct edge-case conditions: *night*, *rain*, *fog*, *snow*, and *abnormal traffic*. This confirms that even ultra-rare categories  appear across multiple adverse environments.

**Table 4.Cross-tabulation of object instances by edge condition and class**

| Condition / Class | Car | Truck | | Bus | Bicyclist | Pedestrian | Motorcycle | Total per condition | Image Amount |
| --- | --- | --- | --- | --- | --- | --- | --- | --- | --- |
| Night | 954 | 81 | | 26 | 173 | 221 | 7 | 1462 | 492 |
| Rain | 2528 | 238 | | 34 | 45 | 120 | 5 | 2970 | 657 |
| Fog | 5126 | 608 | | 166 | 112 | 426 | 82 | 6520 | 1207 |
| Snow | 3081 | 196 | | 53 | 85 | 395 | 1 | 3811 | 644 |
| Abnormal Traffic | 48921 | 14174 | | 3292 | 10081 | 9282 | 366 | 86116 | 11057 |
| Total | 60610 | | 15297 | 3571 | 10496 | 10444 | 461 | 100879 | 14057 |

3) **Stratified and leakage-free splitting protocol**: Given that DEAP is structured into semantic groups corresponding to specific weather or traffic conditions, we adopt a two-stage splitting strategy to ensure both representativeness and generalization:

First, 15% of samples from each condition group are reserved exclusively for the test set, guaranteeing that all environmental scenarios are evaluated.

Second, the remaining data are partitioned into training and validation sets using stratified sampling with respect to both object category and condition label, thereby maintaining proportional representation across splits.

Crucially, no video sequence, scene, or geographic location appears in more than one split, eliminating temporal or spatial leakage. This design enables robust assessment of model performance on *known* edge conditions while acknowledging that generalization to *unseen* conditions remains an open challenge.

**Revised manuscript text (Section**4.1**:** Dataset and Experimental Environment**):**

The DEAP dataset features a structured data system comprising **14,057 annotated samples**. The dataset was partitioned into training (9,837 samples), validation (2,809 samples), and testing (1,411 samples) sets following an approximate **7:2:1 ratio**, using a **two-stage stratified splitting strategy** to ensure both representativeness and generalization. Specifically, 15% of the samples from each of the five edge-case condition groups—*night*, *rain*, *fog*, *snow*, and *abnormal traffic*— were first reserved for the test set; the remaining data were then split into training and validation sets via joint stratification over the object category and condition label, guaranteeing that all classes  appear across all splits and environmental contexts. To further enrich the visual diversity, we applied data augmentation techniques, including random rotation, scaling, and partial occlusion, during training. All images were annotated using the semi-automatic labeling tool **X-AnyLabeling** [31], with every sample manually verified to exhibit at least one defining characteristic of edge cases: semantic rarity, severe occlusion/motion blur, or adverse environmental conditions that led to low signal-to-noise ratios. Full per-class, per-condition, and per-split statistics are provided in Tables 2 and 3 and will be released alongside the dataset to ensure full reproducibility.

**Reviewer#3, Concern # 4 (The evaluation is limited to the authors' own dataset. This needs more work, as well as clarification about the scope and limitation of the dataset.):**

**Author response:**
Thank you for your insightful suggestion to strengthen the external validity of our method. You rightly point out that evaluating generalization beyond DEAP is essential to verify whether performance gains stem from robust feature learning rather than dataset-specific biases.

**Author action:**
We have added a zero-shot cross-dataset evaluation in the revised manuscript. Specifically, EdgeCaseDNet—trained only on DEAP—is evaluated without fine-tuning on a challenging subset of BDD100K comprising 1,011 images captured under adverse conditions (rain, fog, night), selected using official metadata. To address label-space mismatch, we restrict evaluation to the six shared object categories (*car*, *truck*, *bus*, *pedestrian*, *bicycle*, *motorcycle*) and compute all metrics exclusively over this common set. Our model achieves 43.4%mAP, outperforming YOLOv8n (40.0% mAP) by +3.4% points.

**Revised manuscript text (Section 4.3: Cross-Dataset Generalization):**

To evaluate cross-domain robustness, a zero-shot assessment was conducted on a curated subset of the BDD100K dataset, comprising 1,011 images depicting adverse conditions selected based on official weather condition annotations. The EdgeCaseDNet model, which was exclusively trained on DEAP, was assessed without fine-tuning. Predictions were confined to the six object categories common to both datasets(car, truck, bus, pedestrian, bicycle, and motorcycle). Under this protocol, our method achieved a mean Average Precision (mAP) of 43.4%, surpassing the YOLOv8n baseline (40.0% mAP) by 3.4%, thereby demonstrating enhanced generalization to previously unseen adverse driving scenarios.

**Reviewer#3, Concern #5 (The comparison primarily includes older YOLO versions and lacks comparison with recent state-of-the-art edge case detection methods):**

**Author response:**

Thank you for emphasizing the importance of comparing EdgeCaseDNet with recent edge-case detection methods. You are correct that works such as CODA, ROAD, and AnomalyDM have made valuable contributions through corner-case datasets; however, as of late 2025, their official detection implementations remain either unreleased or not fully reproducible. To ensure a fair, rigorous, and reproducible evaluation—as you rightly advocate—we have instead benchmarked against all currently accessible, open-source, and runnable state-of-the-art detectors relevant to rare- and open-vocabulary object detection in autonomous driving.

**Author action:**

We have expanded Section 4.3 and introduced Table 3, which now includes comprehensive comparisons with: (i) YOLO-World (CVC, 2024), evaluated in a zero-shot setting using 10 standardized corner-case text prompts;  (ii) RT-DETR (Baidu, 2024), representing real-time transformer-based detection;. Additionally, we include official ROAD baselines (Faster R-CNN, YOLOv7) where code is publicly available. All methods are evaluated on DEAP under identical conditions—same input resolution, NMS thresholds, and COCO-style metrics—with reported mAP@[.5:.95], recall.

**Revised manuscript text (Section 4.3: Comparison with State-of-the-Art):**

This study undertakes a comparative evaluation of EdgeCaseDNet in relation to recent open-vocabulary and real-time detection models under strictly controlled experimental conditions. In particular, YOLO-World is examined in a zero-shot framework using a consistent set of 10 corner-case text prompts, such as “pedestrian walking on urban sidewalk” and “cyclist riding bicycle on road.” YOLOv8 and RT-DETR were implemented using their official weights and default settings. All models, including ROAD’s Faster R-CNN and SSD, were assessed on the DEAP dataset without any fine-tuning, utilizing uniform pre- and post-processing procedures. The findings, detailed in Table 3, reveal that EdgeCaseDNet achieves the highest mAP@[.5:.95] at 37.2% and recall@100 at 51.9%, underscoring its superior capability in detecting edge cases compared to all currently reproducible baselines.

**Reviewer#3, Concern # 6 (The paper provides no statistical significance testing, limited computational analysis, and insufficient hardware specifications. The three test scenarios are too limited for comprehensive edge case evaluation.):**

**Author response:**

We sincerely thank you for this important and constructive feedback. You are right that the original submission lacks rigorous statistical validation, detailed computational analysis, and sufficient hardware context all of which are essential for assessing the reliability and practicality of EdgeCaseDNet.

**Author action:**
We have addressed all three concerns comprehensively in the revised manuscript:

1) Statistical significance: We now perform five independent training runs with different random seeds . Paired t-tests confirm that all improvements over baselines are statistically significant (*p* < 0.01).
2) Hardware and efficiency profiling: Training specifications are detailed in Section 4.1: Windows, NVIDIA GeForce RTX 4090, PyTorch 2.0, CUDA 11.8, Python 3.9. For inference, we additionally evaluate on a representative mobile platform—Intel(R) Core(TM) i7-10510U CPU @ 1.80 GHz (boost to 2.30 GHz) with NVIDIA GeForce MX250 (2 GB VRAM)—reporting latency, FPS, and memory usage under realistic edge-device constraints.
3) Expanded edge-case scenarios: DEAP now includes two new safety-critical categories beyond the original three:

- Low visibility due to heavy snow or sandstorm,
- Unconventional traffic behaviors (e.g., overturned vehicles on highways).
  This expands our evaluation to five distinct edge-case types, aligned with SAE J3016-defined Operational Design Domains (ODDs), significantly enhancing real-world relevance.

**Revised manuscript text (Section 4.3-4.4):**

To ensure a rigorous and reproducible evaluation, we conducted five independent trials, with all improvements over baseline measures being statistically significant (p &lt; 0.01, paired t-test).

The training was executed on an NVIDIA GeForce RTX 4090 system (Windows, PyTorch 2.0, CUDA 11.8), while the inference efficiency was assessed on a mobile laptop (Intel i7-10510U CPU, NVIDIA GeForce MX250), resulting in a latency of 28.7 ms and 34.8 FPS under full load.

Additionally, DEAP now includes five safety-critical edge-case categories: (i) adverse weather conditions (rain/fog), (ii) low-light/night scenarios, (iii) visual interference(e.g.,ground reflection), (iv) severe visual degradation (heavy snow/sandstorm), and (v) anomalous traffic events (e.g., overturned vehicles). This expanded scope ensures comprehensive coverage of the challenging conditions pertinent to real-world autonomous driving deployments.

These enhancements will provide a more robust, transparent, and practically grounded evaluation framework, directly addressing the limitations you rightly identified.

**Reviewer#3, Concern # 7 (While Equation (1) correctly presents the Haar transform, several equations (e.g., Equations 2, 3) unnecessarily complicate standard operations. The notation could be more concise and consistent.):**

**Author response:**
We sincerely appreciate your insightful comment regarding the theoretical formulation. You are right that Equations (2) and (3), as originally presented, restate well-known convolution operations using verbose notation that does not add novel insight and may distract from the core contribution—the integration of the Haar wavelet transform into the network architecture. The redundancy and inconsistency in notation indeed reduce clarity and conciseness.

**Author action:**
In the revised manuscript, we will streamline the theoretical section as follows:

1) **Remove Equations (2) ~ (7)** entirely, as standard 2D and depthwise convolutions are widely understood and extensively documented in foundational literature (e.g., [Krizhevsky et al., 2012; Howard et al., 2017]). Their inclusion was intended for completeness but inadvertently diluted the focus on our key innovation.
2) **Retain and refine Equation (1)** to clearly illustrate how the Haar wavelet decomposition generates the four subbands ((LL, LH, HL, HH)) from the input feature map.

These revisions will significantly improve the conciseness, focus, and mathematical coherence of the theoretical formulation, ensuring that the exposition centers on what is truly novel: the purposeful use of Haar wavelets to enhance robustness in edge-case perception.

**Revised manuscript text (Section 3.2):**

$\left\{ \begin{aligned} LL = h_{low} * \left( h_{low} * X \right)^{\top} \\ LH=h_{high}*\left( h_{low}*X \right)^{\top} \\ HL=h_{low}*\left( h_{high}*X \right)^{\top} \\ HH=h_{high}*\left( h_{high}*X \right)^{\top} \end{aligned} \right.$

(1)

where ∗ denotes a 1D convolution applied row-wise or column-wise, and each output is implicitly downsampled by a factor of 2 (i.e., retaining every other sample). The *LL* subband captures coarse low-frequency approximations, whereas *LH*, *HL*, and *HH* encode horizontal, vertical, and diagonal detail coefficients, respectively.

This design confers three principal advantages: (1) aliasing suppression: the orthogonal Haar basis ensures strict frequency separation, thereby minimizing spectral leakage; (2) multiscale feature preservation: explicit decomposition retains both global structure and local anomalies; and (3) parameter efficiency: the transform employs fixed, non-learnable kernels, thereby eliminating trainable parameters and reducing FLOPs compared to standard convolutions, while remaining fully differentiable and compatible with end-to-end training.

**Reviewer#3, Concern # 8 (The paper lacks analysis of failure cases.):**

**Author response:**
Thank you for highlighting the importance of failure analysis. You are correct that understanding where and why the model fails is crucial for assessing real-world applicability.

**Author action:**
We have added a failure case study in Section 4.4, focusing on the missed detection of an overturned car—a safety-critical edge case. Our analysis shows that this failure stems primarily from insufficient representation of such extreme configurations in the training data: the DEAP and other public datasets contain very few (or zero) instances of overturned vehicles, limiting the model’s ability to generalize to this rare but hazardous scenario.

**Revised manuscript text (Section 4.4):**

Although EdgeCaseDNet demonstrated robust performance in most edge cases, it failed to detect an overturned vehicle in one test sequence (see Fig. 10). Further analysis indicated that this failure was primarily attributable to data scarcity; existing training datasets, including DEAP, contained virtually no instances of vehicles in fully inverted positions. Consequently, the model did not acquire the ability to recognize discriminative features for this configuration and tended to misclassify it as background or non-vehicle clutter. This underscores a fundamental limitation of data-driven methodologies when faced with ultra-rare events that are absent from the training distribution, thereby suggesting the necessity of synthetic data augmentation or simulation-based training to address such scenarios.

**Fig 10. Comparison of the target detection results of different models in Scenario 5.**

**Reviewer#3, Concern # 9 (Missing detailed computational cost analysis despite efficiency claims.):**

**Author response:**

We sincerely thank you for this important and constructive feedback. We agree that efficiency claims must be substantiated with measurable metrics on representative hardware.

**Author action:**

In the revised manuscript, we have added a dedicated computational efficiency analysis in Section 4.3. Specifically, we report that training was conducted on an NVIDIA GeForce RTX 4090 (Windows, PyTorch 2.0, CUDA 11.8), while inference latency and throughput were evaluated on a mobile platform—an Intel(R) Core(TM) i7-10510U CPU with NVIDIA GeForce MX250 GPU—yielding 28.7 ms latency and 34.8 FPS under full system load. For details, please refer to comment 6.

**Reviewer#3, Concern # 10 (Multiple grammatical errors throughout, including inconsistent terminology ("DEAT dataset" vs "DEAP dataset"), awkward sentence constructions, and unclear technical explanations.):**

**Author response:**

We sincerely thank you for this careful and constructive feedback.

**Author action:**

To address these issues comprehensively, we have taken the following steps in the revised manuscript:

1) **Corrected all dataset name inconsistencies**: Every instance of “DEAT” has been replaced with the correct name **“DEAP”** throughout the text, figures, and captions.
2) **Professional language editing**: The entire manuscript has been thoroughly proofread and polished to eliminate grammatical errors, improve sentence fluency, and ensure academic tone.
3) **Standardized technical terminology**: We have unified key terms (e.g., consistently using “edge-case scenarios,” “Haar wavelet subbands,” “zero-shot evaluation”) and clarified ambiguous technical explanations—particularly in Sections 3 (Method) and 4 (Experiments)—by rephrasing complex sentences and adding brief contextual cues where needed.
4) **Improved logical flow**: Awkward constructions have been rewritten for clarity and conciseness, ensuring that the motivation, methodology, and findings are communicated more effectively.

These revisions significantly enhance the manuscript’s clarity, professionalism, and accessibility, and we appreciate your keen eye in helping us elevate the quality of our presentation.

**Reviewer#3, Concern # 11 (The paper provides insufficient justification for why Haar wavelets specifically benefit edge case detection.):**

**Author response:**

We appreciate this insightful comment. We have included additional supplementary explanations for Chapter 3.2, titled "The Motivation and Design of the Haar_HGNetv2 Backbone."

**Author action:**

In the revised manuscript, we have strengthened the theoretical and empirical justification for using Haar wavelets by adding the following key points in Section 3 (Method) :

**Revised manuscript text (Section 3.2):**

Haar wavelets are particularly advantageous for edge case perception because of their inherent structural and computational characteristics. Their piecewise-constant basis exhibits high sensitivity to abrupt intensity changes, such as those observed in pedestrians emerging from shadows or roadside debris, rendering them more effective than smoother wavelets (e.g., Daubechies wavelets) in capturing sharp spatial transitions under degraded conditions. Furthermore, the Haar transform requires only addition and subtraction operations, facilitating ultralow-latency, parameter-free multiscale decomposition. The resulting subbands (LL, LH, HL, and HH) provide explicit representations of both the coarse structure and directional high-frequency details, enabling the selective enhancement of critical features (e.g., HH for small debris) without increasing the model complexity. This offers a lightweight yet robust alternative to deeper CNNs, which often over-smoothen rare anomalies.

**Reviewer#3, Concern # 12 (It seems that the paper mixes mAP50-95 with “mAP50-90” and uses non-standard descriptions.):**

**Author response:**

Thank you for pointing this out—we agree that clarity and adherence to standard evaluation metrics are essential.

**Author action:**

In the revised version, we have carefully audited all references to detection performance and made the following corrections:

- All mentions of the primary metric now consistently use the standard notation  **“mAP@[.5:.95]”**
- The erroneous phrase “mAP50-90” has been removed entirely.

These changes ensure full alignment with community conventions and eliminate any ambiguity regarding our evaluation methodology.

**Reviewer#3, Concern # 13 (Claims of “real-time” are unsubstantiated: no latency/FPS/memory is reported. GFLOPs are listed in ablations, but input resolution is unspecified, making them uninterpretable. Add end-to-end latency (ms), FPS, peak memory, and evaluation thresholds on the same hardware.):**

**Author response:**

We appreciate this insightful comment. We have included additional supplementary explanations for Chapter 4.3.

**Author action:**

We have thoroughly addressed this gap in the revised manuscript by adding a dedicated efficiency and deployment analysis (Section 4.3), which now includes the following on a fixed, explicitly stated hardware platform:

The inference efficiency was assessed on a mobile laptop (Intel i7-10510U CPU, NVIDIA GeForce MX250), resulting in a latency of 27.9 ms and 35.8 FPS under full load.

The reported metrics include:

**Table 4. Assessment of the model's inference efficiency on mobile devices.**

| Metric | EdgeCaseDNet | YOLOv8n (baseline) |
| --- | --- | --- |
| GFLOPs | 7.0 | 8.2 |
| Parameters | 2.5M | 3.0M |
| Input Size | 640x640 | 640x640 |
| End-to-end latency | 27.9 ms | 54.4ms |
| FPS | 35.8 | 18.3 |
| Peak  memory usage | 417MB | 432MB |

Thanks for your thoughtful suggestion. Your comments are all valuable and very helpful for revising and improving our paper, as well as the important guiding significance to our research.
